# The emotional-behavioral state of Israeli adolescent and young adult females with anorexia nervosa during the COVID19 pandemic

**DOI:** 10.1186/s40337-022-00668-w

**Published:** 2022-10-08

**Authors:** Yaffa Serur, Hadar Dikstein, Tal Shilton, Doron Gothelf, Yael Latzer, Yael Lewis, Adi Enoch-Levy, Itai Pessach, Eitan Gur, Daniel Stein

**Affiliations:** 1 Pediatric Psychosomatic Department, Sheba Medical Center, Safra Children’s Hospital, Tel Hashomer, Israel; 2 Psychiatric Division, Psychiatric Department, Sheba Medical Center, Safra Children’s Hospital, 5265601 Tel Hashomer, Israel; 3 Psychatric Division, Sheba Medical Center, Safra Children’s Hospital, Tel Hashomer, Israel; 4 Sheba Medical Center, Safra Children’s Hospital, Tel Hashomer, Israel; 5 Faculty of Social Welfare and Health Sciences, University of Haifa, Haifa, Israel; 6 Psychiatric Division, Eating Disorders Institution, Rambam Health Care Campus, Haifa, Israel; 7 Shalvatah Mental Health Center, Hod Hasharon, Israel; 8 Center for the Treatment of Eating Disorders and Obesity, Sheba Medical Center, Tel Hashomer, Israel; 9 Department of Psychiatry, Sackler Faculty of Medicine, Tel Aviv University, Tel Aviv, Israel

**Keywords:** Age, Anorexia nervosa, COVID-19, Eating disorders, Females, Pandemic

## Abstract

**Background:**

During the COVID-19 pandemic in Israel, the number of patients with eating disorders (EDs) seeking treatment increased significantly. The present study sought to evaluate whether, during the pandemic (2020–21), patients with anorexia nervosa (AN) would show more ED-related, comorbid, and COVID-19-related symptoms in comparison to a naturalistic control group, and whether differences would be found between adult and adolescent patients with AN. We also examined attitudes to telemedicine use during the pandemic in patients receiving long-distance interventions.

**Methods:**

Using online self-report questionnaires, we assessed general and COVID-19-specific symptoms with a secure digital platform (REDCap^®^) in 36 female adolescents with AN, 35 female adults with AN, and 25 female controls.

**Results:**

Compared with controls, patients with AN showed more symptoms of EDs, anxiety, depression, and post-traumatic stress disorder (PTSD), elevated suicidal ideation, more COVID-related emotional-behavioral disturbances, and lower resilience. Adult patients with AN fared worse than adolescent patients on most of these measures. Adult patients using telemedicine during the COVID-19 pandemic showed fewer positive attitudes toward this treatment than adolescents (telemedicine was offered to all, but used by 18/35 adolescents and 21/36 adults with AN). Last, elevated COVID-19-related symptomatology was correlated with more symptoms of ED, anxiety, depression and PTSD, and with lower resilience.

**Conclusions:**

Our findings suggest that the emotional-behavioral state of Israeli females with AN, particularly adults, was worse during the COVID-19 pandemic in comparison to controls. Many patients did not use telemedicine for their treatment. Adult patients using telemedicine were less satisfied with it than adolescent patients.

## Plain English summary

The recent COVID-19 pandemic has been associated with increased rates of eating disorders in many countries around the globe, including Israel. Our study evaluated whether Israeli patients with anorexia nervosa would fare worse than a control group during the COVID-19 pandemic, and whether adults and adolescents with anorexia nervosa would differ. For this purpose, we administered questionnaires to 71 females with anorexia nervosa—36 adolescents and 35 adults—and to a control group of 25 adolescent and young adult women. We found that females with anorexia nervosa showed not only more eating-disordered and psychiatrically-related symptoms, but also more pandemic-related disturbances than the control group. Among the patients with anorexia nervosa, adults fared worse than adolescents. Fifty-one percent of adolescents and 58% of adults with anorexia nervosa received treatment via telemedicine, although all were offered this treatment. Adults treated with telemedicine showed fewer positive attitudes toward it than adolescents. Last, more disordered eating and other psychiatric symptoms were associated with more COVID-19-specific disturbances. Our findings suggest that the emotional-behavioral state of female Israeli patients with anorexia nervosa is worse than that of control females during the COVID-19 pandemic, especially in adults.

## Background

The recent COVID-19 pandemic has been associated with increased rates of mental illness around the globe, with some countries reporting an estimated threefold rise in depressive symptoms at the start of the pandemic compared to the preceding period [1]. The pandemic, with its consequent quarantines and social isolation, is likely even more challenging for people struggling with emotional problems [2]. Indeed, adults [3, 4] and adolescents [5] with psychiatric problems have been found to show acute emotional reactions to the COVID-19 pandemic, including depression, anxiety, and trauma-related symptoms [3–5].

Emotional-behavioral problems associated with the COVID-19 pandemic may also be found in patients with eating disorders (EDs). Recent surveys of adult patients with anorexia nervosa (AN) and bulimia nervosa (BN) during the COVID-19 pandemic have shown greater reported frequency of food restriction, binge-eating episodes, and purging behaviors, as well as an increase in eating, shape and weight concerns, and drive for physical activity [6–11]. Alongside these symptoms, patients have reported heightened overall anxiety and depression and a reduction in well-being, likely related to economic difficulties, problems with family, and overall social isolation [6–11].

In the only previous study on EDs in Israel during the COVID-19 pandemic, Lewis et al. [12] investigated 51 Israeli patients with different types of EDs in the second and third months of the pandemic (April–May 2020). They found eating disordered symptomatology in the clinical range, moderate depression levels, and mild anxiety and stress. In their sample, 27% were under age 18, but the authors did not differentiate their findings for adolescents versus adults.

In general, findings for adolescents with EDs during the COVID-19 pandemic are more limited. One study found that adolescents presenting for treatment of their ED during the pandemic showed a lower percentage of goal weight, higher rates of self-reported impairments, and a significantly higher likelihood of medical instability and hospitalization compared to those presenting for treatment before the pandemic [13]. Moreover, research showed that almost half of children and adolescents with EDs experienced reactivation of their ED symptoms during the pandemic, including increased symptoms of self-harm and suicide risk among patients with severe EDs [14]. Adolescents with EDs also reported severe health-related anxieties associated with the pandemic [15], alongside increases in depression, anxiety, and trauma-related symptoms [15].

These disturbances likely resulted from the influence of the quarantine-related changes in life circumstances on eating/weight-related behaviors [16] and physical activity [17] of youth with EDs. In contrast to healthy youngsters, during periods such as the COVID-19 pandemic, adolescents with EDs tend to show increased rather than decreased physical activity, and greater restriction rather than greater amounts of food eaten [14].

Moreover, in adolescents with EDs, the greater involvement of their parents in their meals during the lockdown and the inability to go outside and exercise, may further increase their overall distress [16]. Resulting fears of loss of personal control [18] superimposed on an overall rigidity in patients with AN [19], may trigger an increase in weight control behaviors to compensate for this loss [18].

In regular non-pandemic times, management of the medical and psychiatric complications associated with EDs, including low weight, self-harm, and suicidal risk, requires regular face-to-face clinical follow-up visits [20]. However, during the COVID-19 pandemic, such face-to-face assessments could carry a particularly high risk of viral infection and transmission in a vulnerable cohort already compromised by low weight and reduced immunity [20, 21]. Thus, treatment of EDs during the COVID-19 pandemic necessitated adaptations for dealing with home weighing and management of restricting/binging/purging behaviors both in adolescent [16, 20] and adult [11] patients. This required technology-based long-distance interventions from different treatment providers with adolescent and adult patients and various family members, to adequately supervise eating and physical activity [11, 16, 20]. The change from face-to-face treatment to telemedicine might have posed particular difficulties for patients with AN who could become anxious and resistant of home weighing, virtual meal planning, and physical activity supervision [11, 16]. At the same time, lockdown and quarantine experiences appeared to elicit an increase in the use of potentially triggering virtual social media platforms [22].

The first cases of COVID-19 in Israel were reported in February 2020. Soon thereafter, Israel entered its first lockdown (starting March 14, 2020), continuing for around two months. The current study period included two additional lockdowns, in September–October 2020 and December 2020-February 2021. The first COVID-19 immunizations in Israel began at the end of 2020 [23].

With the start of the first national lockdown in Israel, patients were no longer permitted to attend face-to-face treatment unless for emergency care. Indeed, most health services were transferred to telemedicine – a relatively unfamiliar service in Israel for the treatment of EDs. Following the country’s release from that first mandatory lockdown, and for the remainder of the pandemic waves, decisions about face-to-face versus telemedicine provision of ED-related treatment were ad hoc and flexible, as agreed upon by treatment providers and patients/family members [24].

Another impact of the COVID-19 pandemic in many countries around the globe, likely associated with the greater ED-related symptomatic severity at that time, was the greater need for treatment provision [23]. In Israel, the country’s largest health maintenance organization reported 20–100% growth in requests for various ED treatment services during 2020, leading to a 4–12-month increase in waiting times to receive treatment. According to another health maintenance organization, the number of adolescents seeking treatment because of an ED in 2020 increased by 56% [23]. One study found a 2.4-fold increase in the number of adolescents hospitalized in a general pediatric department in Israel in the first year of the COVID-19 pandemic compared to previous years [25].

This trend has also been evident in the substantial growth in the number of ambulatory ED interventions provided between 2019 and 2021 at the Sheba Medical Center, the largest ED treatment center in Israel. As seen on Table 1, a considerable increase emerged in 2021 compared to both 2019 and 2020 in the numbers of treatment sessions, treated ambulatory patients, and new patient admissions into the hospital's specialized ambulatory ED departments for adults and adolescents (despite a small decrease from 2019 to 2020 in the number of patients treated in the adolescent ambulatory service). Telemedicine could have provided a new option in the treatment of patients in both adult and adolescent departments in 2020, considering that it was not used in 2019. All patients in both departments were offered the use of telemedicine treatment during the COVID-19 pandemic. Nonetheless, fewer than half of the given sessions were carried out using telemedicine: 38% of adults and 33% of adolescents in 2020 and 23% of adults and 18% of adolescents in 2021. The decrease in telemedicine usage noted from 2020 to 2021 in both departments likely reflected the lowered pandemic-related restrictions on face-to-face interventions [24].

**Table 1:** Number of sessions and of patients with anorexia nervosa treated by the ambulatory eating disorders (ED) services at the Sheba Medical Center, Tel Hashomer, Israel, between 2019 and 2021

Ambulatory ED services			2019	2020	2021
Adults	Patients	Total	629	618	822
		New	438	404	605
	Sessions	Total	9896	10,841	15,182
		Telemedicine	–	4112	3556
Adolescents	Patients	Total	302	287	398
		New	186	155	214
	Sessions	Total	5521	7539	8600
		Telemedicine	–	2519	1539

To summarize, the COVID-19 pandemic has been associated with an increase in the rate and severity of EDs. To date, most research on EDs during the pandemic has been conducted on adults, and most studies [6–11] but not all [26, 27] included no comparison to control participants. To the best of our knowledge, the current study is one of the first to compare adolescent versus adult patients with AN versus control participants to examine their emotional-behavioral state during the COVID-19 pandemic. (Schlegl et al. [11] has previously shown that adult patients with AN have been more affected symptomatically than adolescents.)

Specifically, we sought to examine ED-related, generalized anxiety, depression, and post-traumatic stress disorder (PTSD) symptoms, suicidal ideation and resilience, as well as pandemic-related symptoms of stress and perceived physical and mental health and functioning. Additionally, we sought to examine the attitudes of adult and adolescent patients with AN to the use of telemedicine treatment. The following were our hypotheses:

1. Compared to control participants during the COVID-19 pandemic, patients with AN would report: (a) more symptoms of ED, generalized anxiety, depression and PTSD, greater suicidal ideation, and lower resilience [as is likely the case also in regular non-pandemic times; see Pollice et al. [28]]; and (b) more pandemic-related symptoms of higher stress and lower physical health, mental health, and functioning.
2. Adult patients with AN would report more comorbid psychiatric symptoms and more pandemic-related symptoms than adolescents with AN, given that the duration of the ED in young adults is likely longer [29].
3. Adult patients with AN will show fewer positive attitudes toward the provision of telemedicine than adolescent patients with AN, likely because adolescents use internet devices more than young adults [30].
4. Pandemic-related health state and functioning will be significantly correlated with ED-related and comorbid psychiatric symptomatology.

## Methods

The study was conducted according to Declaration of Helsinki guidelines and was approved by the Institutional Review Board of the Sheba Medical Center, Tel Hashomer, Israel, as part of a larger research project assessing the COVID-19 pandemic’s impact on its patients with different psychiatric disturbances (Protocol No: SMC-7212-20; June 11, 2020).

### Participants

The study included 96 female participants: 36 adolescents diagnosed with AN, 35 adults diagnosed with AN, and 25 control participants. All 71 participants with AN were treated at the Sheba Medical Center adolescent or adult ED treatment departments, where they received a diagnosis of AN from the departments’ psychiatrists (YS, AEL, EG, DS), who had an adult or child/adolescent psychiatry specialization and who were highly experienced in the diagnosis and treatment of AN. Diagnosis of AN was obtained using a semi-structured interview, the Structured Clinical Interview for *DSM-IV* Axis I Disorders, Patient Edition, Version 2.0) [31], adapted to *the DSM-5* [32] diagnostic criteria. Final diagnoses were confirmed in clinical meetings of the teams of the two departments.

Participants with AN and parents (of minors under age 18) were approached at the medical center by a research assistant (master’s level student in psychology—HD). Control participants included high school students and university students recruited using the snowball method. For all groups, after receiving explanation of the study aims, those agreeing to participate (and parents of minors) received a link to the REDCap^®^ secure digital platform via email or phone and gave written consent (signing their initials) before entering the questionnaires. Participation was voluntary and anonymous, with exit options at any time. From the original pool of 116 participants who responded using the digital platform, 20 were excluded from our analyses because of not completing all questionnaires.

To be noted, the ethical requirements in our facility prevented inclusion of any demographic data other than patients’ age and sex, and also precluded psychiatric assessment of the control group when using the REDCap^®^ platform. Thus, the only demographic parameters assessed in this study were the participants’ age and sex. Accordingly, we had no clinical data about the two groups with AN except for their diagnosis (our inclusion criterion). Likewise, we had no medical or psychological data about the control group. As we could not exclude controls with any psychopathology, including EDs, this group represented a naturalistic rather than a healthy control population.

### Assessment

Participants completed self‐administered anonymous questionnaires online using the REDCap^®^ platform, and responses were saved on a secure server at the Sheba Medical Center.

#### Eating attitudes test-26 (EAT-26) [33]

This widely used standardized self-report assesses symptoms and concerns characteristic of disordered eating. Its 26 items relate to body image disturbances, concerns about eating, and behaviors related to dieting, over-eating, and purging. Each item is scored on a 6-point Likert scale, with higher scores indicating greater disturbance. A score of ≥ 20 indicates the likelihood of disordered eating. The EAT-26 has been previously shown to successfully differentiate Israeli patients with EDs from non-ED controls [34]. Internal consistency (Cronbach alpha) for the EAT-26 in this study is α = 0.95.

#### Generalized anxiety disorder 7 (GAD-7) [35]

This self-report is used as a screening tool and severity measure for assessing symptoms compatible with generalized anxiety disorder. The GAD-7 consists of 7 items rated on a 4-point Likert scale, with higher scores indicating greater anxiety. A score of ≥ 10 is considered a reasonable cutoff point for identifying persons with this disorder [35]. The GAD-7 has previously been used for patients with EDs [36], and its Hebrew translation has been validated for Israeli populations [37]. Internal consistency of the GAD-7 in this study is α = 0.91.

#### Patient health questionnaire-9 (PHQ-9) depression module [38, 39]

This 9-item depression module from the PHQ is rated on a 3-point Likert scale, with higher scores indicating greater depression. The PHQ-9 has previously been used for individuals with EDs [36], and its Hebrew translation has been validated for Israeli populations, including patients with EDs [40]. Internal consistency of the PHQ-9 self-report in this study is α = 0.92.

#### Ask suicide-screening questions (ASQ) tool [41, 42]

This brief 5-item tool for identifying individuals at risk for suicide consists of four yes/no questions about different contexts of suicidal ideation in general, in the past few weeks, and in the past week. An additional fifth question, about having suicidal thoughts right now, is asked only if any of the four previous questions is answered positively—to identify acute suicidal risk in clinical populations. For the current research, we used two subscales of suicidal ideation deriving from the ASQ: existence and severity. Existence of suicidal ideation was scored 1 (answering “yes” to at least 1 of the 5 ASQ items) or was scored 0 (answering “no” to all items). Severity of suicidal ideation was scored 0–2: No suicidal ideation—“no” to all items (scored 0), mild suicidal ideation—“yes” to 1–2 ASQ items (scored 1), and severe suicidal ideation—“yes” to 3–5 ASQ items (scored 2). The original ASQ has been validated for both adolescents and adults [41, 42] but not for patients with EDs. It was translated to Hebrew by an NIMH team (https://www.nimh.nih.gov/sites/default/files/documents/research/researchconducted-at-nimh/asq-toolkit-materials/asqtranslations/asq_hebrew_translation.pdf). The current ASQ adaptation to two self-reported subscales has not been validated. Internal consistency of the 5-item ASQ in this study is α = 0.78.

#### Primary care post-traumatic stress disorder screen for DSM-5 (PC-PTSD-5) [43]

This 5-item screening questionnaire was designed to identify individuals with probable PTSD in primary care settings [43]. It has a score range of 0–5, with higher scores indicating greater severity of PTSD symptoms. The standard cutoff score of ≥ 3 is used to classify probable PTSD. The Hebrew version of the PC-PTSD-5 has been validated and used previously in Israeli populations [44, 45]. It has not been previously used in patients with EDs. Internal consistency of the PC-PTSD-5 self-report in this study is α = 0.83.

#### Pandemic‐related stress factors (PRSF) [46]

This 18-item inventory for assessing stress, recommended by a previous Israeli study for the COVID-19 pandemic context [47], is compiled from items shown to be pertinent in previous research in Japan on the N1H1 pandemic [48]. Participants rate items along a 4‐point Likert‐type scale from 0 (never) to 3 (always), with higher scores indicating greater COVID-19 related stress.

In the present study, we excluded 9 of the original 18 PRSF items because they were not considered suitable for adolescents. In our modified 9-item PRSF scale, 2 items tapped fear of being infected, 2 items tapped lack of adequate knowledge about COVID-19, 2 items tapped feeling protected from infection (e.g., by the Israeli health system), and 3 items tapped emotions related to different aspects of COVID-19 stress. Internal consistency of the modified PRSF self-report in this study was α = 0.62.

#### Connor-Davidson resilience scale-10 (CD-RISC-10) [49]

This 10-item self-report assesses participants' resilience potential following stressful and traumatic events. Participants rate items on a 5-point Likert scale from 0 (not true at all) to 4 (true nearly all the time), with higher scores indicating greater resilience. The Hebrew translation of this scale has been validated and used in Israeli populations [44]. Internal consistency of the CD-RISC-10 in this study is α = 0.89.

#### Specific COVID-19 related questions

Participants were asked to rate the following four pandemic-related questions: (a) How do you describe your state of health compared to other people of your age? (on a 5-point Likert scale, from far below average to far above average); (b) Do you feel that your mental state changed during the COVID-19 crisis? (on a 5-point Likert scale, from considerably deteriorated to considerably improved); (c) How do you describe your overall functioning? (on a 7-point Likert scale, from not functioning to excellent functioning); and (d) Did you experience symptoms associated with the COVID-19 virus? (rated as no vs. 3 different positive possibilities, i.e., experiencing symptoms and not checked, or negative testing/positive testing). For all these questions (on current health relative to age group, current mental state relative to pre-pandemic, current overall functioning, and viral symptoms), higher scores indicated more COVID-19-related difficulties. These questions were based on previous studies of our group [50].

#### Use of telemedicine during the pandemic

As noted earlier, all patients in both departments were offered telemedicine treatment during the COVID-19 pandemic. Most patients who did not opt for telemedicine were treated in face-to-face sessions (except during the first lockdown). It might be speculated that these patients were likely to receive fewer treatment sessions than those treated with telemedicine, because of inherent difficulties of our center in maintaining regular face-to-face treatment in both departments during the pandemic, but this was not evaluated.

Those patients with AN in this study who did use telemedicine for their treatment (18/35 adults and 21/36 adolescents) were asked to rate three items on a 4-point Likert scale from never to always: Did you feel that treatment using the internet was (a) effective, (b) helped you, and (c) left you satisfied? High intercorrelations among the three items enabled their combination into one total score (efficacy–help: *r* = 0.81; efficacy–satisfaction: *r* = 0.81; help–satisfaction: *r* = 0.72). Higher scores indicated more favorable perceptions of telemedicine. Internal consistency of this combined telemedicine scale was α = 0.93. The COVID-19 telemedicine-related satisfaction questions were constructed by the research and clinical teams of the adolescent and adult ED departments of the Sheba Medical Center.

### Statistical analyses

We used analysis of covariance (ANCOVA) to compare group differences on the six continuous variables (EAT-26, GAD-7, PHQ-9, PC-PTSD-5**,** PRSF, CD-RISC-10), as well as on the participants' health state, mental state, functioning, and telemedicine use during the pandemic. Time elapsed between the first COVID-19 lockdown and participants’ date of online survey completion served as a covariate. Age served as an additional covariate in the comparison between patients with AN and control participants. Chi-square analyses were used for between-group comparisons on the two categorical variables (ASQ and experiencing COVID-19 symptoms). The first comparison was between patients with AN and controls. The second comparison was between adolescent and adult patients with AN. To adjust for the possibility of type-I error due to multiple comparisons, we used the false discovery rate correction. The adjusted *p* values are presented in the text and tables.

## Results

### Demographic data

#### Patients with AN versus controls

**Fig. 1:**
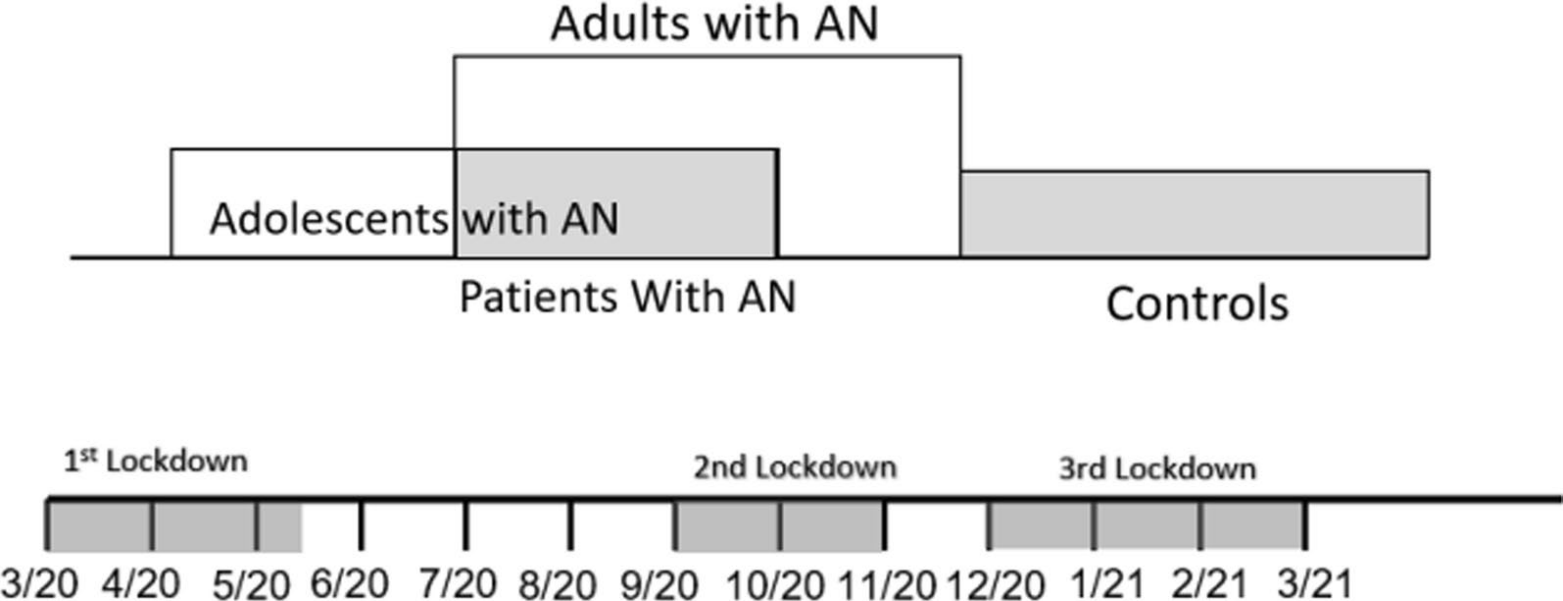
Recruitment of patients with anorexia nervosa (AN) and controls during the COVID-19 pandemic in Israel

The age range of patients with AN was 13–26 years, with a mean of 17.44 years (*SD* = 3.17 years). The age range of the control group was 13–25 years, with a mean of 20.00 years (*SD* = 4.07 years). Thus, patients and controls differed significantly by age (*F* = 10.310, *p* = 0.002). Also, the mean time elapsed between the beginning of the first COVID-19 lockdown in Israel (March 14, 2020) and participants’ online survey date was 197.37 days (*SD* = 62.81) for patients with AN and 338.76 days (*SD* = 28.91) for controls. This difference was significant (*F* = 117.308, *p* = 0.0001). Most patients with AN were surveyed around the second lockdown (September–October 2020), whereas most of the controls were surveyed around the end of the third lockdown (December 2020-February 2021). This is described in Fig. 1.

#### Adolescents vs. adults with AN

Among the patients with AN, the adolescents’ age range was 13–17.5 years, with a mean of 14.94 years (*SD* = 1.74 years), and the adults’ age range was 18–26 years, with a mean of 20.01 years (*SD* = 2.02 years). This difference was significant (*F* = 104.907, *p* < 0.001). The mean time elapsed between the beginning of the first COVID-19 lockdown and participants’ online survey date was 175.6 days (*SD* = 59.57) for adolescents with AN and 219.74 days (*SD* = 58.85) for adults with AN (see Fig. 1). Although most patients of both age groups were assessed during the second lockdown (September–October 2020), this difference was significant (*F* = 9.87, *p* = 0.02).

#### Treatments for patients with AN

Despite being offered telemedicine options, 15 of the 36 adolescents (42%) and 17 of the 35 adults (49%) were not treated with telemedicine during the COVID-19 pandemic. No differences were found in any of variables included in the study between patients treated with telemedicine and patients treated otherwise (mostly with face-to-face interventions). Thus, all analyses were conducted without controlling for treatment mode.

### Outcome measures

#### Differences between patients with AN and controls

Table 2 summarizes the differences between patients with AN and control participants, with age and time elapsed (from the first lockdown to the assessment interval) serving as covariates. Patients with AN showed greater ED-related pathology on the EAT-26, greater anxiety on the GAD-7, greater depression on the PHQ-9, greater severity of PTSD symptoms on the PC-PTSD-5, greater COVID-19-related stress on the PRSF, and lower resilience on the CD-RISC-10 compared to controls. Patients with AN also rated their health state, mental state, and functioning during the pandemic as lower in comparison to control participants (see Table 2).

**Table 2:** Differences between patients with AN and controls in psychometric variables

	Patients with AN (*n* = 71)	Controls (*n* = 25)	*F*	*p* (adjusted)*
Eating disorder symptoms (EAT-26)	40.39 (18.31)	8.56 (9.77)	108.17	< 0.0001
Generalized anxiety disorder (GAD-7)	11.04 (5.58)	5.96 (5.00)	24.61	< 0.0001
Depression (PHQ-9)	14.82 (7.19)	5.84 (4.74)	32.90	< 0.0001
Posttraumatic stress disorder (PC-PTSD-5)	2.79 (1.91)	1.04 (1.36)	33.57	< 0.0001
Pandemic-related stress (PRSF)	12.53 (4.01)	10.64 (3.95)	5.14	< 0.05
Resilience (CD-RISC-10)	19.70 (8.56)	30.04 (6.72)	17.57	< 0.0001
Functioning during COVID-19 pandemic	4.39 (1.64)	5.76 (1.12)	28.31	< 0.0001
Health condition during COVID-19 pandemic	2.58 (0.71)	3.80 (0.76)	27.66	< 0.0001
Mental state during COVID-19 pandemic	1.77 (0.86)	2.40 (0.81)	19.14	< 0.0001

^*^adjusted according to the false discovery rate correction; age and time between the first lockdown and survey date are covariates

*AN* Anorexia nervosa; *EAT-26* Eating attitudes test-26; *GAD-7* Generalized anxiety disorder 7; *PHQ-9* Patient health questionnaire-9 (Depression Module); *PC-PTSD-5* Primary care post-traumatic stress disorder screen for *DSM-5*: *PRSF* Pandemic‐related stress factors; *CD-RISC-10* Connor-Davidson resilience scale-10

When assessing for the existence of suicidal ideation on our modified ASQ, significantly more patients with AN had evidence of suicidal ideation than controls (χ^2^_(2) _= 27.766, *p* < 0.0001). Specifically, 69% of the patients with AN versus 12% of the controls answered positively to at least one ASQ item. When assessing for the severity of suicidal ideation on our modified ASQ, a significant between-group difference emerged (χ^2^_(2) _= 24.239, *p* < 0.0001). The differences between the two groups are summarized in Table 3. Specifically, almost one third (29%) of the patients with AN had severe suicidal ideation, compared to only 4% of the control participants.

**Table 3:** Between-group differences in the severity of suicidal ideation using the ASQ

	*n*	Suicidal ideation			χ^2^(2)	*p*
		None (%)	Mild (%)	Severe (%)		
All patients with AN	71	31	40	29	24.239	< 0.0001
Controls	25	88	8	4		
Adults with AN	35	17	37	46	10.438	= 0.005
Adolescents with AN	36	44	42	14		

Severity of suicidal ideation is described with percentages

*AN* Anorexia nervosa; *ASQ* Ask suicide-screening questions tool

No significant differences were found between patients with AN and control participants in their reported rates of experiencing COVID-19 viral symptoms (χ^2^_(2)_ = 0.437, *p* = 0.509). Thus, 31% of the patients with AN and 24% of the controls reported having experienced COVID-19 symptoms.

#### Differences between adult and adolescent patients with AN

Table 4 summarizes the differences between adult and adolescent patients with AN, with time elapsed since the first lockdown serving as a covariate. Significant between-group differences emerged for most but not all variables. Thus, adult patients with AN showed significantly higher scores in eating-related disturbances (EAT-26), anxiety (GAD-7), depression (PHQ-9), severity of PTSD symptoms (PC-PTSD-5), and COVID-19-related stress (PRSF) compared to adolescents. Adult patients with AN also rated their mental state and functioning during the pandemic as lower than the adolescent AN group (see Table 4). No significant differences emerged between the two patient groups for resilience (CD-RISC-10) or for ratings of health state.

**Table 4:** Differences between adult and adolescent patients with AN in the psychometric variables

	AN- adults (*n* = 35)	AN-adolescents (*n* = 36)	*F*	*p* (adjusted)*
Eating disorder symptoms (EAT-26)	48.40 (12.62)	32.61 (19.74)	8.07	< 0.05
Generalized anxiety disorder (GAD-7)	13.51 (4.68)	8.64 (4.68)	11.20	< 0.05
Depression (PHQ-9)	17.83 (6.09)	11.89 (7.03)	10.58	< 0.05
Posttraumatic stress disorder (PC-PTSD-5)	3.63 (1.61)	1.97 (1.85)	9.93	< 0.05
Pandemic-related stress (PRSF)	13.91 (4.25)	11.19 (3.31)	6.96	< 0.05
Resilience (CD-RISC-10)	18.86 (9.36)	20.53 (7.75)	0.33	0.564
Functioning during COVID-19 pandemic	3.80 (1.77)	4.97 (1.27)	4.87	< 0.05
Health condition during COVID-19 pandemic	2.46 (0.78)	2.69 (0.62)	1.40	0.270
Mental state during COVID-19 pandemic	1.43 (0.65)	2.11 (0.91)	7.39	< 0.05
Telemedicine-related effectiveness, help and satisfaction	2.14 (.50) (*n* = 18)	2.68 (0.80) (*n* = 21)	5.931	< 0.05

^*^Adjusted according to the false discovery rate correction; time between the first lockdown and survey date is a covariate

*AN* Anorexia nervosa; *EAT-26* Eating attitudes test-26; *GAD-7* Generalized anxiety disorder 7; *PHQ-9* Patient health questionnaire-9 (Depression Module); *PC-PTSD-5* Primary care post-traumatic stress disorder screen for *DSM-5*, *PRSF* Pandemic‐related stress factors; *CD-RISC-10* Connor-Davidson resilience scale-10

Significantly more adult patients with AN had evidence of suicidal ideation compared to adolescent patients according to our modified (yes/no) ASQ subscale (χ^2^_(2)_ = 7.766, *p* = 0.020). Thus, 83% of the adult patients had evidence of suicidal ideation in comparison to 56% of the adolescent patients. When assessing for the severity of suicidal ideation on our modified ASQ, a significant difference emerged between the two groups (χ^2^_(2) _= 10.438, *p* = 0.005). The differences between the two groups are summarized in Table 3. Most importantly, almost half (46%) of the adult patients with AN had evidence of severe suicidal ideation, compared to only 14% of the adolescent patients.

No significant difference emerged between adult and adolescent patients with AN in their self-reported rates of COVID-19 physical symptoms (χ^2^_(2)_ = 0.351, p = 0.55). Thus, 34% of the adult patients and 28% of the adolescent patients reported experiencing COVID-19 symptoms.

Last, as seen in Table 4, among those patients with AN receiving telemedicine treatment, adults showed fewer positive attitudes toward the telehealth experience compared to adolescents.

#### Correlations among the COVID-19-specific parameters and the general psychiatric parameters

As seen in Table 5, most correlations among the study variables were significant. In general, less favorable ratings of specific COVID-19-related parameters were significantly associated with greater disturbance in the general psychopathological parameters, including higher ED-symptomatology, anxiety, depression, and PTSD symptoms, and lower resilience (for two of the four pandemic-related scales).

**Table 5:** Correlations among COVID-19-related and general psychometric parameters

General parameters	Pandemic-specific indices			
	Pandemic-related stress (PRSF)	Functioning during COVID-19	Health condition during COVID-19	Mental state during COVID-19
Eating disorder symptoms (EAT-26)	0.267*	− 0.492***	− 0.191 ^*ns*^	− 0.379**
Generalized anxiety disorder (GAD-7)	0.429***	− 0.493***	− 0.226 ^*ns*^	− 0.385**
Depression (PHQ-9)	0.390**	− 0.572***	− 0.264 ^*ns*^	− 0.429***
Posttraumatic stress disorder (PC-PTSD-5)	0.331**	− 0.530***	− 0.371***	− 0.570***
Resilience (CD-RISC-10)	− 0.239*	0.413***	0.176^ ns^	0.083^* ns*^

^*^p < .05; **p < .001; ***p < .0001; *ns* = not significant

*EAT-26* Eating attitudes test-26; *GAD-7* Generalized anxiety disorder 7; *PHQ-9* Patient health questionnaire-9 (Depression Module); *PC-PTSD-5* Primary care post-traumatic stress disorder screen for *DSM-5*; *PRSF* Pandemic‐related stress factors; *CD-RISC-10* Connor-Davidson resilience scale-10

## Discussion

To the best of our knowledge, this is one of the first studies comparing the emotional-behavioral state of adolescent and adult patients with AN and control participants during the COVID-19 pandemic. In addition, we examined differences in the attitudes of adult and adolescent patients with AN toward the use of telemedicine for their treatment.

### Differences between patients with AN and controls

In line with our first hypothesis, during the COVID-19 pandemic, patients with AN have shown elevated levels of ED-related symptomatology, anxiety, depression, PTSD symptomatology and COVID-related stress, as well as lower resilience, in comparison to control participants (see Table 2). These pandemic-time differences between patients with AN and control participants were expected, as they characterize also ordinary non-pandemic times [28]. Nonetheless, several pandemic-related explanations may account for these between-group differences. First, in comparison to controls, the tendency of patients with AN to adhere to stricter, more rigid routines [19] may reduce their ability to adapt to lifestyle changes [51] such as those required during the COVID-19 pandemic. Second, problems in the delivery of routine treatments during the pandemic, such as regular assessment of food intake, weight, and overall medical condition, as well as difficulties in adapting to the use of telemedicine, may further increase the overall disturbances of patients with AN [7, 8, 14, 20, 52–54], Third, during lockdowns, patients with AN may experience greater distress because of decreases in physical activity, increased involvement of family members with their eating, and isolation from their friends [16, 52].

Alongside the greater disturbances shown by patients with AN on their ED and comorbid parameters, significantly more of these patients showed evidence of suicidal ideation, specifically severe suicidal ideation, in comparison to the controls (see Table 3). Adverse life stressors [55], including the presence of infectious disease outbreaks [56–58] like the COVID-19 pandemic, might increase suicidal ideation. In line with our results, a recent study in China [56] found that people with preexisting mental disorders revealed a higher prevalence of suicidal ideation during the COVID-19 pandemic in comparison to the general population. Moreover, pandemic-related quarantine, unemployment, and increased psychological stress were found to be particularly relevant in increasing the risk and severity of suicidal ideation in those participants with mental disorders [56].

Moreover, our study found higher rates of PTSD symptoms and pandemic-related stress and lower levels of overall functioning, physical health, and mental state during the COVID-19 pandemic in patients with AN compared to control participants (see Table 2). Other studies also found that patients with EDs show more PTSD symptomatology than controls [26, 27]. It is of note that our patients with AN demonstrated elevated PTSD symptomatology even though most of them were surveyed around 6–7 months after the COVID-19 pandemic began (during the second lockdown), whereas most control participants were surveyed about 10–12 months after the start of the pandemic (during the third lockdown; see Fig. 1). Thus, although the control participants could potentially have been more negatively influenced by longer exposure to the pandemic’s effects, they fared better than the participants with AN.

A possible reason for the greater emotional-behavioral disturbance of people with AN in our study in comparison to controls could be their significantly lower resilience (see Table 2). Resilience is most often defined as the ability to cope with and recover from setbacks, adapt well to changes, and persevere in the face of adversity [57, 58]. It has been described as a protective factor, where individuals with high resilience show a lower tendency to develop PTSD symptoms [57, 58]. Moreover, resilience may predict the individuals’ ability to improve when presented with adverse life difficulties [59]. In line with our findings, previous studies have also found lower resilience in patients with AN compared to control participants, associating this finding with the presence of interoceptive confusion, interpersonal difficulties, negative self-image, and ineffectiveness in patients with AN, as well as with an influence of the ED-related pathology per se [60, 61].

### Differences between adult and adolescent patients with AN

In line with our second hypothesis, during the COVID-19 pandemic, adult patients with AN showed significantly more ED-related symptoms, anxiety and depression, as well as greater severity of PTSD symptoms and pandemic-related stress in comparison to adolescent patients with AN. In addition, the adult group rated their functioning and mental state during the COVID-19 pandemic as lower than the adolescent group (see Table 4). Also, significantly more of the adult patients had evidence of suicidal ideation, specifically severe suicidal ideation, in comparison to adolescent patients with AN (see Table 3).

Some studies in trauma [62], although not all [63], have found that young adults may report the highest prevalence of current/recent traumatic and other stressful life events in comparison to other age groups [62]. Factors putatively increasing the influence of traumatic events in young adults may include their potentially less favorable living-working conditions in terms of less supportive family relationships and social support, problems with occupation, and residence changes [64].

In the case of EDs during the COVID-19 pandemic, adolescents with AN are usually confined at home together with their parents, whereas young adults who might have already left their original family, can find themselves alone, with less support from parents, partners, or friends [65, 66]. Prior studies during routine non-pandemic times have shown that families and other support systems are required to assist patients with EDs with their treatment [67]. Yet, some families of patients with EDs do not function that well [16, 68], and adolescents with EDs may find the greater involvement of their parents in their eating because of being more at home during the lockdown distressing [16]. Nonetheless, this involvement may bring the problems associated with the ED to the foreground [16], increasing the possibility for earlier professional intervention [16]. It is of note that these possible explanations are only speculative, as we have no data about the families of our participants.

Another factor to be considered regarding the greater vulnerability of adults compared to adolescents with AN involves the duration of illness. As a greater percentage of the young adult group have likely started their illness during their adolescent years rather than in early adulthood [29], their longer duration of illness in comparison to the adolescent group may be associated with an overall less favorable outcome [69, 70].

### Differences between adult and adolescent patients with AN in their attitudes to telemedicine

The COVID-19 pandemic necessitated many modifications to the treatment of patients with EDs [20]. Studies reported rapid increases in the usage of telehealth within ED programs for adolescents and adults as a result of the pandemic [11, 20, 71], as shown also in Israel in general [23] and in our medical center (see Table 1). Nevertheless, the use of telemedicine during the COVID-19 pandemic presented several challenges and drawbacks for patients with EDs, including problems with weighing and supervising meals and physical activity, privacy concerns, and difficulties in ensuring accurate communication and treatment consistency [72, 73].

In the present study, more adolescents and adults with AN were treated with face-to face treatment than with telehealth during the COVID-19 pandemic, despite the accessibility of telemedicine and the likelihood that face-to-face interventions would be less frequently available. Similarly, one study found that three quarters of patients with EDs preferred face-to-face therapy over telemedicine [74], and in another study, only a third of patients with AN used videoconference therapy and/or online interventions [11]. Moreover, in a previous Israeli study [12], the majority (68%) of adult patients with different types of EDs stated that they would not choose to continue online therapy given the option. These findings stand in contrast to studies in Israeli adults of different age groups with other medical disorders, showing that over 60% used telemedicine health services [75, 76]. The current results are also different from the positive attitudes expressed by adult patients with AN toward long-distance treatments provided during regular non-pandemic times [77]. Perhaps this relates to the fact that during routine times, telemedicine may be chosen as one of multiple possible treatment options, whereas during the COVID-19 pandemic it was more a necessity than a choice.

The reluctance of patients with AN to adopt telehealth treatment during the COVID-19 pandemic may be linked to reduced motivation to recover [73] and to specific ED-related characteristics such as elevated rigidity, sensitivity, social anxiety, and avoidance, as well as to body-image issues that could affect patients’ willingness to show themselves on camera over the internet [19, 78–81]. In comparison to patients with other types of EDs, people with AN may express the greatest difficulty and dissatisfaction with telemedicine during the pandemic, owing to specifically heightened sensitivity to privacy concerns, particularly with respect to eating and weighing considerations [79].

In line with our third hypothesis, adult patients with AN were less satisfied overall with the use of telemedicine compared to adolescent patients (see Table 4). One factor potentially decreasing the satisfaction of adult AN patients with the use of telemedicine may be their increased inclination toward self-management [82]. By contrast, adolescents find it difficult to manage by themselves in times of crises [14]. Second, adolescents are likely more familiar with the use of internet-based communication devices than adults [30]. Third, adolescents with AN may specifically benefit from the distancing effect of telemedicine because of the high influence of comorbid social anxiety issues in this age [83], and because of their concern with face-to-face interactions, often considered overwhelming [84]. Last, people with more severe psychological distress tend to use telemedicine less [73]; in our study, adults had more severe ED-related and comorbid symptoms than adolescents.

### Correlations among the specific COVID-19-related parameters and the ED and comorbid psychiatric parameters

In line with the fourth hypothesis, our study showed that less favorable self-reports on COVID-19-related state (in terms of functioning, health, mental state, and severity of pandemic-related stress) were significantly associated with greater ED-symptomatology, anxiety, depression, and post-traumatic symptomatology (see Table 5). In addition, lower resilience was found to correlate with greater severity of general and COVID-19-related symptoms.

To the best of our knowledge, previous studies on patients with EDs during the COVID-19 pandemic have not assessed correlations between COVID-19 and ED/comorbid psychiatric-related disturbances. Our findings support previous research showing an association between reactions to stressful situations in general (not pandemic-related) and elevated psychiatric symptomatology, especially in individuals with preexisting psychopathology [85, 86]. Also, in the general population, studies during the COVID-19 pandemic have shown that elevated depressive and anxious symptoms are associated with more general and COVID-19-specific stress [87]. Moreover, a longitudinal study during the pandemic [88] has found that despite a reduction over time in the severity of COVID-19-related stress, persons with elevated depression continue to report greater stress.

### Limitations and strengths

The current findings should be regarded as preliminary and addressed with caution because of several limitations. First, we had no baseline data about the status of the participants before the pandemic. Second, the cross-sectional design of our study without follow-up precluded conclusions about causality. Third, this was a relatively small convenience sample, treated in one specific treatment center, rather than representing a randomly selected population. Moreover, the sample included only female patients without male representation. Fourth, we used self-rating questionnaires, and our new scale measuring satisfaction toward telemedicine among Israeli participants with AN was not previously validated. Finally, ethical requirements in our facility prevented inclusion of any demographic data other than the patients’ age and sex when using the REDCap^®^ platform, and also precluded psychiatric assessment of the control group.

Our study nevertheless offers some important advantages. This hypothesis-generated research utilizes appropriate tools for the diagnosis of AN as well as comprehensive multidimensional assessment of general and pandemic-related psychopathology and functioning. Most of the questionnaires have been previously used in the study of populations in crisis. We have changed two instruments, by adapting the original adult version of the PRSF to better fit adolescent populations and by modifying the ASQ to assess both the existence and severity of suicidal ideation rather than assessing current clinical suicidal risk. It is of note that the ASQ changes have not yet been validated. Yet, the adapted ASQ and adapted PRSF have significantly differentiated between patients with AN and controls, and between adults and adolescents with AN, in a similar direction to that of the other study questionnaires. Another strength is that we have considered covariant factors in the analyses such as the different periods of evaluation between the groups. Finally, although our sample is relatively small, the between-group differences are mostly highly robust.

## Conclusions, clinical implications, and directions for future research

The main findings of our study are that during the COVID-19 pandemic, patients with AN fare less well than controls, and adult patients with AN fare less well than adolescents with AN. The greater vulnerability of patients with AN might be related, in part, to their lower resilience. The greater vulnerability of adults with AN may be related, in part, to their probable longer duration of illness. In addition, at least in Israel, not many patients with AN use telemedicine during the pandemic, and adults are less satisfied with telemedicine than adolescents.

Future studies should be prospective and longitudinal, including pre-pandemic data and larger samples of female and male patients with different types of EDs, to verify our preliminary findings. This would enable assessment of the potentially harmful influence of the COVID-19 period on patients with AN over time, in comparison to the pre-pandemic era.

## Data Availability

The datasets used and/or analyzed during the current study are available from the corresponding author upon request. Participants completed self‐administered anonymous questionnaires online using the digital REDCap^®^ platform, and responses were saved on a secure server at the Sheba Medical Center, Tel Hashomer, Israel.

## Abbreviations

AN: Anorexia nervosa
ED: Eating disorder
PTSD: Post-traumatic stress disorder
EAT-26: Eating attitudes test-26
GAD-7: Generalized anxiety disorder 7
PHQ-9: Patient health questionnaire-9
PC-PTSD-5: Primary care post-traumatic stress disorder (PTSD) screen for *DSM-5*
PRSF: Pandemic‐related stress factors
CD-RISC-10: Connor-Davidson resilience scale-10
